# Socio-economic status, racial composition and the affordability of fresh fruits and vegetables in neighborhoods of a large rural region in Texas

**DOI:** 10.1186/1475-2891-10-6

**Published:** 2011-01-18

**Authors:** Richard A Dunn, Joseph R Sharkey, Justus Lotade-Manje, Yasser Bouhlal, Rodolfo M Nayga

**Affiliations:** 1 Department of Agricultural Economics, Texas A&M University, College Station, TX, USA; 2 Program for Research in Nutrition and Health Disparities, School of Rural Public Health, Texas A&M Health Science Center, College Station, TX, USA; 3 Department of Agricultural Economics and Agribusiness, University of Arkansas, Fayetteville, AR, USA

## Abstract

**Background:**

Little is known about how affordability of healthy food varies with community characteristics in rural settings. We examined how the cost of fresh fruit and vegetables varies with the economic and demographic characteristics in six rural counties of Texas.

**Methods:**

Ground-truthed data from the Brazos Valley Food Environment Project were used to identify all food stores in the rural region and the availability and lowest price of fresh whole fruit and vegetables in the food stores. Socioeconomic characteristics were extracted from the 2000 U.S. Census Summary Files 3 at the level of the census block group. We used an imputation strategy to calculate two types of price indices for both fresh fruit and fresh vegetables: a *high variety *and a *basic *index; and evaluated the relationship between neighborhood economic and demographic characteristics and affordability of fresh produce, using linear regression models.

**Results:**

The mean cost of meeting the USDA recommendation of fruit consumption from a high variety basket of fruit types in our sample of stores was just over $27.50 per week. Relying on the three most common fruits lowered the weekly expense to under $17.25 per week, a reduction of 37.6%. The effect of moving from a high variety to a low variety basket was much less when considering vegetable consumption: a 4.3% decline from $29.23 to $27.97 per week. Univariate regression analysis revealed that the cost of fresh produce is not associated with the racial/ethnic composition of the local community. However, multivariate regression showed that holding median income constant, stores in neighborhoods with higher percentages of Black residents paid more for fresh fruits and vegetables. The proportion of Hispanic residents was not associated with cost in either the univariate or multivariate analysis.

**Conclusion:**

This study extends prior work by examining the affordability of fresh fruit and vegetables from food stores in a large rural area; and how access to an affordable supply of fresh fruit and vegetables differs by neighborhood inequalities. The approach and findings of this study are relevant and have important research and policy implications for understanding access and availability of affordable, healthy foods.

## Introduction

There are well-documented disparities in both dietary intake and diet-related health conditions among racial, ethnic and socio-economic groups in the United States [1]. Understanding the causes of these differences has become a major focus of research and policy [2]. Physical environment has been associated with numerous health behaviors and outcomes [3,4], leading many to hypothesize that difference in eating behavior originate from differences in access to healthy food options [5-9]. For example, consumption of fruit and vegetables is recommended through the Dietary Guidelines for Americans [10], but these foods are often not easily accessible by racial and ethnic minority groups in large urban centers or populations in rural areas [1,11-19]. Along with reduced access, fresh fruit and vegetables may also be less affordable to rural populations and racial/ethnic minority groups, which would also contribute to nutrition-related disparities [13,16,20-22].

Attempts to study the relationship between neighborhood characteristics and the affordability of healthy food options, such as fresh fruits and vegetables, have utilized data from predominantly urban areas [11,12,21,23-27]. Studies that do consider rural populations have focused on differences in affordability across store types, rather than across the characteristics of residents [16,26]. As a result, very little is known about how the demographic and economic composition of rural neighborhoods is related to the affordability of healthy food items. Therefore, the aim of this article is to investigate whether stores located in rural neighborhoods with lower socio-economic status or with higher proportions of African-American and Hispanic residents charge more for fresh fruit and vegetables. Previous findings on the relationship between the affordability of healthy food options and neighborhood economic status using in urban settings are mixed. While some find a positive relationship between socio-economic status [24,25], others have reported that there is no statistically significant differences [23,27].

We examine fruits and vegetables since their consumption is associated with positive health outcomes, they account for a substantial portion of the USDA Thrifty Food Plan market basket, and previous research on their availability, affordability and consumption provide a basis from which we can compare our results. We focus on fresh fruit items since they account for roughly 81% of total non-juice fruit consumption. Fresh vegetables account for 52% of total vegetable consumption that is neither dehydrated or chipped (i.e. potato chips) [28].

Our approach is novel in several respects. Our information on the price of fresh fruits and vegetables comes from ground-truthed data collected by taking an on-site census of food stores in a large regional area. Moreover, this rural region in central Texas is home to a socio-economically and demographically diverse population. This differs from previous work on rural affordability, which has generally considered smaller geographic areas that are racially homogenous or used only a sample of stores. Finally, we handle missing prices through an imputation strategy based on economic theory; specifically the decision of a profit-maximizing store owner to stock a particular item for sale. Previous methods that used simple mean imputation procedures, e.g. replacing missing prices with the mean price over similar store types, have raised concerns that this method may lead to biased results [21].

## Methods

### Setting

The seven contiguous counties of the Brazos Valley are situated between the Dallas and Houston metropolitan areas. The region is home to 300,000 residents, of which 51.4% reside in one of six rural counties, which cover a land area of 4,466 m^2 ^[29]. These six counties [Burleson, Grimes, Leon, Madison, Robertson and Washington) define our region ("rural BV") and exhibit a great deal of economic and demographic diversity. For example, county population ranges from 13,379 to 32,034; median household income from $28,964 to $35,852, percent of African American residents 10.1% to 22.9%, and percent Hispanic from 10.9% to 18.9%.

In this analysis, we define neighborhoods according to census block groups (CBG), since these are the smallest unit of census geography for which the detailed "long-form" social and economic data from the census are tabulated [17,23]. There is no agreement on the proper definition of a neighborhood in the food environment literature [30], and while some studies have defined neighborhoods by CBG [23,25], others have utilized the larger geography of census tracts [5,11,31]. As a CBG typically contains 250 to 550 housing units and approximately 600 to 3,000 people, the relatively low population density associated with our rural sample suggests that the CBG is an appropriate definition of neighborhood. The socioeconomic characteristics of the 101 CBG in the rural BV region were extracted from the 2000 decennial census Summary Files 3 (SF-3). The characteristics included are median household income, the proportion of Black residents and the proportion of Hispanic residents.

### Brazos Valley Food Environment Project (BVFEP)

As part of the BVFEP, trained observers enumerated all food stores and food service places by driving all Interstates, US Highways, Texas State Highways, Texas Farm-to-Market Roads and other major thoroughfares to locate and geocode (i.e., assign geographic coordinates to specific locations) all stores that could sell food items [17]. The geographic coordinates (latitude and longitude) for the specific location of each food store were determined by a camera-based GPS. Geographic position was measured in front of each food store with a Bluetooth Wide Area Augmentation System-enabled portable GPS receiver after at least 4 satellite signals were detected; the World Geodetic System 1984 datum was used [17]. Using the geographic coordinates and 2000 U.S. Census Summary File 3, ArcGIS (Version 9.2, Environmental Systems Research Institute) assigned the appropriate CBG for each food store. As previously published, the BVFEP used ground-truthed methods in a two-stage approach to determine the location of all food stores and the availability of fresh produce [17,32]. Identification and surveying occurred between September 2006 and June 2007. Food stores were classified into several categories: supercenter, supermarket, grocery store, convenience store, dollar store, mass merchandiser, and pharmacy. The BVFEP identified 1 supercenter, 12 supermarkets, 11 grocery stores and 140 convenience stores across the rural BV region. Investigators entered all food stores with an extensive list of food items on a tally sheet in order to catalogue which items were sold and at what price [32]. Based on interviews local residents and nutrition professionals [32], ten types of fresh fruit (apples, avocado, bananas, berries, grapes, mango, melons, oranges, peaches and pears) and eleven types of fresh vegetables (broccoli and cauliflower, carrots, corn, green beans, leafy greens, lettuce, okra, onions, potatoes, squash and tomatoes) were included in this catalogue. The following information was recorded: whether each type (e.g. apples) was available for purchase; the number of varieties of each type of fruit or vegetable available for purchase; and the lowest-priced variety of each type of fruit or vegetable. Because in-store prices were posted in several forms--per item, per ounces, per pound--all prices were later transformed or recalculated into a uniform price per pound. To do so when prices were posted per item, surveyors weighed the items using a sensor scale. The price of food items that were either not sold or not displayed were recorded as missing. Surveyors did not purposefully interact with store managers or employees during the data collection process.

Although there are many fruit and vegetable varieties that were not included in the BVFEP, the 9 of the 10 fruits used here (mango was omitted from the subsequent analysis given its very limited availability and low consumption share) accounted for 80% of consumption and expenditure according to the Fresh Look Marketing data. Lemons, limes and tangerines are the most commonly consumed fruits not included. The ten of the 11 vegetables used in the analysis (okra was omitted given its very limited availability and low consumption share) account for 72% of all fresh whole vegetable expenditure and 75% of consumption. The most common varieties not included are celery, cucumber, mushrooms and peppers.

### Price Imputation

In previous studies, missing prices have either been ignored so that mean prices are calculated only over the sample of stores that sell a particular item or set of items [25] or they have been imputed by taking the mean price over the stores that do sell the item [21,24,31,33]. These imputation strategies may be misguided since stores that do not sell a particular item are likely not comparable to the average store that does [21]. Instead, one could view the decision of a store-owner not to stock an item for sale as the result of profit-maximizing behavior. The price that consumers are willing to pay for the missing type is below the cost that a store owner faces to offer the type for sale. Nevertheless, there is still some reservation price that would lead the store owner to stock the item. Thus, the proper price for missing types is this unobserved *shadow price *and intuitively, it should be higher than the mean observed price. Ignoring the underlying reasons for missing price information may not be benign to the purpose at hand. If economically disadvantaged neighborhoods suffered from both low availability and affordability of fresh produce, then using the mean price calculated from stores in economically advantaged neighborhoods would understate the true relationship between economic status and affordability.

Alternatively, one may take a statistical perspective and assume that over some period of time, all stores eventually stock all types of fresh produce. Since inventory and prices are only observed once for each store, it is possible that unobserved types were recently sold or will be sold again soon. The goal is then to reasonably estimate these unobserved prices given the observable price data. Both the economic and statistical perspectives suggest using a price imputation strategy that takes advantage of the observed prices in each store. For example, a store that sells apples and bananas above the mean price found in others stores would likely charge an above average price for berries, as well. Therefore, in the current study the price of each fruit or vegetable item was first estimated as a linear function of the store type, the county in which the store was located and the prices of the most common fruit or vegetable types--apples, oranges and bananas for fruit and onions, potatoes and tomatoes for vegetables. The coefficient estimates from these regressions were then used to impute values for the missing prices of other types [34]. For comparison, we also repeat our analysis using mean imputation.

### Price indices

The actual and imputed prices (the former when available, the latter when missing) were then used to calculate two types of price indices for both fresh fruit and fresh vegetables: a *high variety *and a *basic *index. The high variety index includes the full set of items, while the basic index includes only the most common items (apples, bananas and oranges for fruit and carrots, lettuce, onion, potatoes and tomatoes for vegetables). Such a distinction may be relevant for policy-makers, for example, if the intention of a particular program is to provide assistance to increase the amount of fruit and vegetable consumption with little importance attached to the variety of types consumed.

Each index is calculated as the weighted mean price per pound multiplied by the recommended number of pounds consumed per week for a representative family of two adults and two children from the most recent USDA Thrifty Food Plan: 24.5 pounds of fruit and 31.5 pounds of vegetables. The Thrifty Food Plan expects that fruit and vegetable consumption will also come from a mix of sources (e.g. fresh whole, frozen, canned, dried, etc.), but the choice of multiplicative factors affects the magnitude of the subsequent coefficient estimates, but not their statistical significance. If only half of total fruit consumption should come from fresh whole items, then the appropriate adjustment is to either halve the coefficient estimate or reinterpret it as biweekly expenditure. The weights are equal to the consumption shares calculated for the Dallas metropolitan area from Fresh Look Marketing, Inc. (Chicago, Illinois) and represent all supermarkets (sales of at least $2 million) with about 70% of all commodity volume (ACV) in the Dallas market (Timothy Richards, personal communication).

### Statistical analysis

A linear relationship between these explanatory variables and each price index was then estimated using multivariate ordinary least squares regression models. Therefore, each observation was a store, with the store-level price index being the dependent variable and the set characteristics of the CBG in which the store is located being the explanatory variables. As is common in regression analysis, median household income was taken in its natural logarithm so that its coefficient estimate is interpreted as the effect of a 100% increase in variable.

It is important to note that the number of observations available for the regression analysis was relatively small. Even with imputation of missing prices, only 21 stores posted enough price information to calculate a high variety price index for fruit and 23 stores posted enough price information to calculate a high variety price index for vegetables. Since the typical issues associated with small sample sizes--large standard errors and low powered hypothesis tests--are exacerbated by multi-collinearity between explanatory variables, we calculated variance inflation factors (VIF) to assess collinearity [35].

Because multiple stores can be located in a single CBG, robust standard errors clustered at the CBG-level were calculated [36]. Statistical significance was determined using an alpha of 5%, but given the relatively small sample size, we were also interested in results that approach significance, i.e. alpha of 10%. Values presented are means ± SD.

## Results

Among the 25 stores that sold at least 3 fruit items (1 supercenter, 12 supermarkets, 10 grocery and 2 convenience), apples, oranges, avocado and banana were the most commonly found (Table 1 provides both the number of stores with price data by item along with the proportion of stores doing so). On a per weight basis, bananas were the cheapest fruit type, while berries were the most expensive. Among the 32 stores that sold at least 4 vegetable items (1 supercenter, 12 supermarkets, 11 grocery stores and 8 convenience stores), carrots, lettuce, onions, potatoes and tomatoes were the most common. Potatoes were the least expensive vegetable type, while green beans were the most expensive.

**Table 1:** Price Availability, Conditional Mean Price and Consumption Shares of Fresh Fruit and Vegetables Types

	**Stores with non-missing price**^**1**^	**Mean price**^**2**^	Min	Max	**Consumption share**^**4**^
		($)	($)	($)	(%)

***Fruits***					
apples	23(92%)	1.10 ± 0.32	0.61	1.99	11.7
avocado	22(88%)	2.34 ± 1.07	0.74	5.47	9.1
bananas	22(88%)	0.52 ± 0.10	0.33	0.69	33.8
berries	15(60%)	3.00 ± 0.61	2.00	3.99	6.2
grapes	19(76%)	1.77 ± 0.54	0.89	2.79	8.9
melon	18(72%)	0.75 ± 0.24	0.33	1.12	18.2
oranges	23(92%)	0.93 ± 0.36	0.33	1.49	7.7
peaches	13(52%)	1.68 ± 0.34	1.27	2.29	2.6
Pears	11(44%)	1.11 ± 0.43	0.35	1.69	1.8
*All types*^3^	8(32%)				100
***Vegetables***					
carrots	23(72%)	1.01 ± 0.46	0.49	1.98	7.8
Corn	13(41%)	0.97 ± 0.39	0.45	1.82	5.7
cruciferous	16(50%)	0.92 ± 0.32	0.32	1.49	3.8
Green beans	10(31%)	1.52 ± 0.50	0.99	2.79	2.1
greens	15(47%)	1.00 ± 0.31	0.70	2.01	1.1
lettuce	25(78%)	0.81 ± 0.34	0.49	1.98	7.7
onions	24(75%)	0.97 ± 0.39	0.39	1.99	16.4
potatoes	24(75%)	0.62 ± 0.42	0.30	2.39	33.8
tomatoes	24(75%)	1.34 ± 0.43	0.69	2.39	4.8
squash	17(53%)	1.18 ± 0.40	0.50	1.88	17
*All types*^3^	10(31%)				100

^1 ^For fruit, sample is all stores selling at least three types: n = 25. For vegetables, sample is all stores selling at least four types: n = 36. The proportion of stores with non-missing price among those selling at least three fruit types and at least 4 vegetable types in parentheses.

^2 ^Conditional means (mean price over stores with non-missing price information) are reported ± SD. For example, the mean price of apples in the 23 stores with non-missing prices is $1.10 per pound with a standard deviation of $0.32. The lowest observed price in these 23 stores is $0.61 per pound, while the highest observed price is $1.99 per pound.

^3^*All types *summarizes the number of stores selling all types of fruits or vegetables, respectively.

^4^Consumption share of each type from Fresh Look Marketing, Inc for Dallas market.

There were 25 stores that sold at least 3 types of fruit; however, 22 stores posted the requisite information--the prices of apples, oranges and bananas--to calculate the basic variety price index (1 supercenter, 12 supermarkets, 8 grocery stores, and 1 convenience store). Similarly, of the 32 stores that sold at least four types of vegetables, 23 posted the requisite price information to calculate the basic vegetable price index (1 supercenter, 12 supermarkets, 8 grocery stores and 2 convenience stores). The mean cost of meeting the USDA recommended level of fruit consumption from a high variety basket of fruit types in our sample of stores was just over $27.50 per week (Table 2). In contrast, relying on only the three most common fruits lowered the weekly expense to just under $17.25 per week, a reduction of 37.6%. The effect of moving from a high variety to a low variety basket was much less when considering vegetable consumption: a 4.3% decline from $29.23 to $27.97 per week.

**Table 2:** Summary statistics of produce availability, store types and CBG characteristics

	n	**Mean**^**1**^	Min	Max
*Price indices, $*				
High variety fruit	21	27.58 ± 4.24	19.93	37.89
Basic fruit	22	17.22 ± 2.89	10.16	23.63
High variety vegetable	23	29.23 ± 6.70	17.19	48.19
Basic vegetable	23	27.97 ± 7.93	15.88	53.24
*Store characteristics, %*				
Proportion grocery stores	23	56.5		
Proportion grocery stores	23	34.8		
Proportion convenience stores	23	8.7		
*CBG characteristics*				
Median family income, $	23	30,083 ± 7,29	14,400	50,547
Proportion Black, %	23	21.2 ± 16.1	29.7	74.5
Proportion Hispanic, %	23	16.5 ± 9.7	49.0	37.1

Notes: Price indices are the cost of purchasing the recommended weekly servings of fruits and vegetables according to the USDA Thrifty Food Plan for a representative household of 2 adults and 2 children from fresh, whole items: 24.5 pounds (11.1 kg) of fruit and 31.5 pounds (14.3 kg) of vegetables). Not all price indices can be calculated for all stores because of variation in which prices for individual goods are available. Store types and CBG characteristics calculated over the set of stores for which a basic fruit index or a basic vegetable index or both could be calculated. Means are reported ± SD.

^1 ^Conditional means (mean price over stores with non-missing price information) are reported ± SD.

Although there are 101 CBG in the six counties of the rural Brazos Valley region, the overwhelming majority either does not have a food store located within their boundaries or the only food stores are convenience stores that do not sell fresh fruits or vegetables. Nevertheless, the 23 food stores with sufficient price information to be included in the subsequent analysis are located across diverse neighborhoods. The median household income at the level of the CBG was highly variable, ranging from $14,400 to $50,500. On average, African American comprised 21.2% of the CBG population, while Hispanics accounted for 16.5%. There were also several minority-majority CBG in which more than 50% of the population was either African American or Hispanic. Indeed, the CBG were these 23 food stores are located (Table 2) appear slightly more diverse than the region in general.

Multivariate regression analysis of the store-level price indices on CBG characteristics (Table 3) revealed that stores in CBG with higher median household incomes tended to charge more for fresh fruits and vegetables, holding racial composition equal. The coefficient on the logarithm of median household income was positive and statistically significant (P < 0.05) for both vegetable price indices. For example, every ten percent increase in the CBG median household income was associated with an increase of $1.39 (P < 0.05) in the high variety index and $1.61 (P < 0.05) in the basic index. The coefficient estimate was also positive and was statistically significant at the 10% level for the high variety fruit index, implying that a 10% increase in median income was associated with a $1.02 (P = 0.089) increase.

**Table 3:** Coefficient estimates from OLS multivariate regression models

	**Multivariate regression**^**1**^			
	
	*Fresh fruit*		*Fresh vegetables*	
	
	**High variety**^**2**^	**Basic**^**3**^	**High variety**^**2**^	**Basic**^**3**^
	
	b (SE)	b (SE)	b (SE)	b (SE)
Median family income^4^	10.15* (5.99)	-0.12 (3.49)	13.87* (6.94)	16.12* (8.11)
Proportion African American	0.149* (0.087)	0.010 (0.055)	0.178 (0.112)	0.230 (0.123)*
Proportion Hispanic	0.050 (0.098)	0.043 (0.086)	0.009 (0.158)	0.030 (0.161)

Observations	21	22	23	23

R^2^	0.19	0.02	0.14	0.15
Maximum VIF	2.21	2.44	2.37	2.37
Mean VIF	1.79	1.93	1.89	1.89

^1^Coefficient estimates from multivariate linear regression (when all explanatory variables enter simultaneously). Robust standard errors clustered at CBG level in parentheses below coefficient estimates. *P < 0.1 **P < 0.05 ***P < 0.01

^2^High variety indices include all produce types listed in Table 1.

^3^Basic fruit index only includes apples, bananas, and oranges. Basic vegetable index only includes carrots, lettuce, onion, potatoes and tomatoes.

^4 ^Taken in natural logarithm.

In addition, stores in CBG with greater proportions of Black residents tended the charge more for fresh produce, holding income equal. The coefficient estimates on the proportion of Black residents were positive for all four indices and were statistically significant at the 10% level for two. Holding everything else constant, a 10 percentage point increase in the proportion of African American residents was associated with an increase of $1.49 (P = 0.085) in the high variety fruit index and $2.30 (P = 0.062) in the basic vegetable index. In contrast, the coefficient on the proportion of Hispanic residents was typically close to zero and never approached significance (the smallest P-value is 0.621). Finally, the mean VIF was at or below 2.0 in each regression and the maximum was never larger than 2.5, indicating that collinearity between explanatory variables is not problematic.

The results using multivariate analysis differ quite substantially from the univariate analysis (Table 4). For example, stores in higher income areas do not charge more for the basket of fresh fruits and vegetables when differences in racial composition are not simultaneously controlled. Similarly, stores in neighborhoods with higher proportions of African American resident do not tend to charge more for fresh fruits and vegetables. The difference between the multivariate and univariate analyses is explained by the negative relationship between the proportion of African American residents in a neighborhood and the median household income.

**Table 4:** Coefficient estimates from OLS univariate regression models

	**Univariate regression**^**1**^			
	
	*Fresh Fruit*		*Fresh Vegetables*	
	
	**High variety**^**2**^	**Basic**^**3**^	**High variety**^**2**^	**Basic**^**3**^
	
	b (SE)	b (SE)	b (SE)	b (SE)
Median family income^4^	3.21 (4.83)	0.01 (2.11)	5.74 (3.72)	6.19 (4.45)
Proportion African American	0.039 (0.071)	0.008 (0.037)	0.021 (0.079)	0.043 (0.081)
Proportion Hispanic	-0.022 (0.119)	0.040 (0.082)	-0.079 (0.167)	0.125 (0.181

Observations	21	22	23	23

^1^Coefficient estimates from univariate linear regression (when explanatory variables enter one at a time). Robust standard errors clustered at CBG level in parentheses below coefficient estimates. *P < 0.1 **P < 0.05 ***P < 0.01

^2^High variety indices include all produce types listed in Table 1.

^3^Basic fruit index only includes apples, bananas, and oranges. Basic vegetable index only includes carrots, lettuce, onion, potatoes and tomatoes.

^4 ^Taken in natural logarithm.

We also repeated our multivariate regression analysis replacing price indices with the prices (imputed price if missing) of individual fruit and vegetable items. Among fruits (Table 5), the price of avocado was strongly related to both income and the racial/ethnic composition of the neighborhood, whereas the remaining fruit types were very weakly associated with either. This result likely explains why the *R^2 ^*for the high variety index reported in Table 4 is much larger than that for the basic index, which does not incorporate avocado. It is worth noting that this finding cannot be explained by the imputation process, as the price of avocado did not need to be imputed at any of the 21 stores used in the analysis. Although the relationship between household income and affordability of fruit (and between racial/ethnic composition and affordability) is principally the results of one item, avocado is nonetheless an important ingredient in local Tex-Mex cuisine and accounts for a non-trivial proportion of total fruit consumption, 9.1%.

**Table 5:** Association between neighborhood characteristics and the price of individual fresh fruit items^1^

		*Fruit*				
		apples	avocados	bananas	berries	grapes
Median family income^2^		0.50 (0.45)	3.78*** (1.37)	-0.16 (0.10)	0.21 (1.04)	0.54 (0.60)
Proportion African American		0.005 (0.006)	0.066*** (0.019)	-0.001 (0.002)	-0.009 (0.015)	0.007 (0.008)
Proportion Hispanic		-0.001 (0.007)	0.032* (0.018)	-0.004 (0.003)	-0.003 (0.014)	0.007 (0.016)

Observations		21	21	21	21	21
R-squared		0.09	0.48	0.17	0.11	0.04
		Melons	Oranges	Peaches	Pears	

Median family income^2^		0.09 (0.38)	-0.03 (0.40)	-0.30 (0.49)	-0.06 (0.55)	
Proportion African American		0.001 (0.005)	-0.004 (0.007)	0.002 (0.007)	0.002 (0.007)	
Proportion Hispanic		0.000 (0.005)	0.001 (0.010)	-0.002 (0.011)	0.005 (0.016)	

Observations		21	21	21	21	
R-squared		0.01	0.02	0.08	0.02	

^1^Coefficient estimates from multivariate linear regression. Robust standard errors clustered at CBG level in parentheses below coefficient estimates. *P < 0.1 **P < 0.05 ***P < 0.01

^2 ^Taken in natural logarithm.

Among vegetables (Table 6), green beans actually exhibit a negative relationship to both income and the proportion of Black or Hispanic residents, but more common items like onions, lettuce, potatoes and tomatoes display qualitatively similar results to those presented in Table 4 for the price indices. Although the coefficient estimates in these regressions are not statistically significant, estimating a statistically significant association between a particular neighborhood characteristic and an index of prices when the individual price relationships are not statistically significant should not be troubling. Since stores that sell high prices for one type likely charge a higher price for similar items, i.e. a positive covariance between prices, the sum of variances will be less than the variance of the sum. Contrary to being troubling, this is evidence that the intuition underlying our imputation strategy was reasonable. It also suggests that studies of affordability should report results using both aggregate and disaggregated measures of affordability, since aggregate prices can obscure important behavior among individual items, while results for individual goods may not adequately account for the positive covariance of prices within a given store.

**Table 6:** Association between neighborhood characteristics and the price of individual fresh vegetable items^1^

		Vegetables				
		carrots	corn	cruciferous	greens	green beans
Median family income^2^		-0.16 (0.44)	0.03 (0.53)	0.26 (0.30)	-0.30 (0.33)	-2.05** (1.03)
Proportion African American		0.009 (0.007)	0.002 (0.008)	-0.002 (0.005)	-0.004 (0.005)	-0.032* (0.018)
Proportion Hispanic		0.005 (0.009)	0.017 (0.014)	0.014* (0.008)	-0.011 (0.008)	-0.038** (0.019)

Observations		23	23	23	23	23
R-squared		0.15	0.16	0.15	0.11	0.22
		Lettuce	Onions	Potato	Squash	Tomato

Median family income^2^		1.11 (0.67)	0.51 (0.54)	0.48 (0.39)	1.08*** (0.41)	0.62 (0.49)
Proportion African American		0.009 (0.008)	0.003 (0.008)	0.008 (0.006)	0.006 (0.007)	0.009 (0.007)
Proportion Hispanic		0.008** (0.004)	0.008 (0.014)	-0.010 (0.007)	0.009 (0.010)	0.003 (0.009)

Observations		23	23	23	23	23
R-squared		0.31	0.06	0.13	0.26	0.07

^1^Coefficient estimates from multivariate linear regression. Robust standard errors clustered at CBG level in parentheses below coefficient estimates. *P < 0.1 **P < 0.05 ***P < 0.01

^2 ^Taken in natural logarithm.

Finally, we repeated the analysis using mean imputation (not reported). In this particular application, the choice of imputation method did not affect coefficient estimates, but it may nevertheless be influential in other settings.

## Discussion

The goal of this article was to describe the relationship between the prices charged by stores for healthy food items and the characteristics of the surrounding communities. We found that holding racial composition constant, stores located in neighborhoods with higher income residents charge more for fresh fruits and vegetables. This is consistent with previous results in other urban settings [24,25]. For fruit, much of the difference in cost between stores was accounted for by differences in the price of one item, avocado. For vegetables, however, the relationship between neighborhood income and price was present for many of the most common types.

A positive relationship between the affluence of surrounding communities and the cost of fresh produce (holding racial composition constant) can be explained by a number of forces. First, fresh produce is a normal good, i.e. households purchase more fresh fruits and vegetables as their incomes increase. Some of this increase can be attributed to a general increase in all purchases as income goes up. It is also possible that households with higher incomes shift their consumption of fruits and vegetables toward fresh items, instead of canned or frozen versions. Of course, these explanations are not mutually exclusive and could be occurring concurrently. Regardless of the mechanism through which income causes demand for fresh produce to increase, basic economic theory predicts that as demand for fresh produce increases, the price of produce will also increase.

A second explanation is that fresh produce is not a homogenous product. Quality is also a normal attribute so that as incomes increase, individuals attempt to replace low-quality items with higher quality version. Hence, differences in prices between stores may reflect differences in the freshness of items.

Finally, stores in rural areas may face less price competition. In densely populated areas, higher demand for fresh produce may not lead to appreciably higher prices since there are many stores competing for the same set of potential consumers. Since rural areas typically exhibit low population densities combined with large distances between population centers, the number of stores that are reasonably accessible to residents will be smaller, reducing the amount of competition.

Holding the median household income constant, stores located in neighborhoods with higher proportions of African American residents also tended to charge a higher price for fresh produce items. Part of the explanation may be that greater proportions of African Americans reside in CBG where there is greater access to small grocery and convenience stores, where prices are higher than charged in the larger supermarkets and supercenters [16,17,32]. In contrast, the proportion of Hispanic residents did not exhibit a statistically significant relationship to affordability. As is well-documented, African Americans in the United States suffer from higher rates of nutrition-related illness such as diabetes and are much more likely than Whites to be obese [37,38]. This is also evident in the Brazos Valley region, where one study found that the obesity rate for Blacks is 46.4% compared to 32.0% for Whites [39]. The affordability of fresh produce may be one factor that contributes to the disparity.

Univariate analysis revealed that failing to control for income and racial composition simultaneously can influence coefficient estimates, and thus policy implications. For example, our multivariate regression results suggest that a policy that sought to reduce income inequality between neighborhoods with high and low proportions of Black residents could drive up the price of fresh produce, so that in real (purchasing power) terms, neighborhoods with higher proportions of Black residents would remain relatively disadvantaged. This clearly important effect is missed in the univariate analysis.

### Strengths

In addition to providing a fuller description of affordability differences by economic status and racial/ethnic composition in rural areas, the current paper also makes several methodological improvements over previous work. First, our data is from a census of food outlets that utilized ground-truthing. While ground-truthing methods have been used elsewhere, they often are based on just a sample of stores [23,31], cover a relatively small geographic area [21,27] or are employed in areas with limited economic or racial/ethnic heterogeneity [26,40,41]. In our application, all stores in a large rural region that is economically and demographically diverse are present in the dataset.

Second, the analysis confronts the common problem of missing prices using a price imputation strategy that is more firmly grounded in economic and statistical theory than has previously been employed, addressing a potential source of bias [21,34]. Although results with our regression imputation strategy were similar to those using mean imputation in this particular analysis, in other contexts ignoring the profit maximizing behavior of store-owners may not be benign. More generally, researchers in this literature should take greater care with their imputation decisions by explicitly recognizing the underlying assumptions inherent in any method and checking the robustness of their results.

### Limitations

The analysis suffers from several limitations. First, the number of supermarkets and grocery stores in the rural Brazos Valley region is relatively small and future work should consider canvassing a larger area to increase the precision of estimates. Doing so could not only increase the number of observations in an analysis similar to the one undertaken here, but also allow for separate regressions for urban and rural areas. There are obvious trade-offs between completeness and breadth of coverage and small sample sizes are shared with previous work that employed ground-truthing [16,20,40,41]. While suggesting that larger data collection efforts be undertaken, we also acknowledge that the cost associated with completing a census of food stores should not be underestimated

Second, we are unable to translate differences in local affordability into differences in purchasing behavior. If transportation costs are low, then local affordability may not influence actual purchasing or consumption decisions. This could be the case if individuals lived in rural areas, but commuted to an urban center for work. The six counties in the rural BV region are circumjacent to Brazos County, which has a population of almost 150,000 concentrated in the cities of College Station and Bryan and home to Texas A&M University. In future work, we wish to consider how the affordability of neighborhood food prices influences the decision of where to shop, and the activity/travel patterns of rural residents.

A closely related issue is translating differences in affordability into differences in eating behavior. If higher prices do not affect consumption patterns, then attempts to lower the cost of fresh produce may lead to overall gains in welfare, but will not influence nutrition-related health outcomes. This may be particularly salient in explaining racial and ethnic disparities, as the association between neighborhood socio-economic status and consumption of fruits and vegetables is stronger for Blacks than Whites [1]. Again, this is an area of future research.

Third, it must be acknowledged that fresh whole items from food stores are not the only source of fruit and vegetables, though as stated previously, the majority of fruit and vegetable consumption is in the form of fresh whole items. In food stores, fruit and vegetables can also be purchased in frozen, canned, dried and juiced forms. Additionally, fruit and vegetable consumption may also occur in restaurant settings. Since the nutritional value of consumption likely varies by the form consumed, the affordability of these different options, both in absolute terms and relative to each other, is also worthy of future study.

Fourth, sales shares were not available for the stores in our sample, and thus we were unable to weight store-level observations in the regression analysis. Future data collection effects should do so in order to account for differences in the relative importance that each store plays in the actual purchasing decisions of households. Alternative methods include weighting stores by a combination of cash registers in service and hours of operation [25], but this information was not collected during surveying.

Finally, while our imputation strategy allows us to calculate a hypothetical measure of affordability for stores that do not sell all items, these stores may typically exhibit limited availability. We are unable to document a possible variation in produce prices due to seasonal variation. Researchers and policy makers should keep both aspects--availability and affordability--in mind when considering improvements in the food environment.

## Conclusion

Despite these limitations, this study extends prior work by examining the affordability of fresh fruit and vegetables from food stores in a large rural area; and how access to an affordable supply of fresh fruit and vegetables differs by neighborhood inequalities. The approach and findings of this study are relevant and have important research and policy implications for understanding access and availability of affordable, healthy foods. Access to a good variety of affordable healthy foods, such as fruit and vegetables, can play a pivotal role in the nutritional health of rural families. Many of these families live in socioeconomically-deprived neighborhoods; many have a low household income, are unemployed, older, or lack access to a vehicle. In order for rural families to be food secure and have access to fruit and vegetables, food resources need to be available and affordable in local stores.

## Competing interests

The authors declare that they have no competing interests.

## Authors' contributions

RAD, JRS, and RN designed research; RAD and JRS conducted research; RAD, JL-M., and YB analyzed data; RAD and YB were responsible for the final analyses; RAD and JRS wrote the paper. All authors read, commented on, and approved the final manuscript.
